# Palpitations: Evaluation and management by primary care practitioners

**DOI:** 10.4102/safp.v64i1.5449

**Published:** 2022-02-24

**Authors:** Indiran Govender, Kamelia K. Nashed, Selvandran Rangiah, Sunday Okeke, Olga M. Maphasha

**Affiliations:** 1 Department Family Medicine and Primary Health Care, Faculty Health Sciences, Sefako Makgatho Health Sciences University, Pretoria, South Africa; 2 Department of Family Medicine, Faculty of Health Sciences, University of KwaZulu-Natal, Durban, South Africa; 3 Department of Family Medicine, Faculty of Medicine, University of Pretoria, Pretoria, South Africa; 4 Department of Family Medicine, University of Pretoria and Kalafong Provincial Tertiary Hospital, Pretoria, South Africa

**Keywords:** palpitations, ECG, arrhythmia, chest pain, tachycardia

## Abstract

Palpitations are a common, non-specific presenting complaint in primary healthcare and emergency departments. Palpitations are mostly a symptom of benign underlying disease but a sign of life-threatening conditions. Importantly, palpitations are a symptom and not a diagnosis, and cardiac causes are the most concerning aetiology. Clinicians should seek to identify the underlying cause. History and physical examination are important in the assessment of patients with palpitations, and the use of a 12-lead electrographic (ECG) monitor on presentation is the gold standard of diagnosis. If the aetiology cannot be determined, an ambulatory Holter 24–48-h monitor can be used. Treatment and follow-up of patients presenting with palpitations as the main complaint will depend on the aetiology and investigation findings. Patients with palpitations accompanied by dizziness, excessive fatigue, or chest pains should receive adequate acute care aiming to stabilise their condition before referring to a higher level of care.

## Introduction

Palpitations are defined as the awareness of abnormal heartbeat, rapid pulsation or irregular beating of the heart.^1^ They are often described by the patient as a rapid fluttering, skipping or pounding sensation in the chest or neck.^1,2,3^ The symptom may reflect a cardiac or non-cardiac cause or a high catecholamine state.^4^

Palpitations are a frequent symptom in the general population and one of the most common presentations to general practice.^2,3,5^ They are the second most common reason for primary healthcare referrals to cardiologists.^3^

Palpitations are associated with long-term morbidity with a substantial proportion of patients reporting concern and anxiety despite the exclusion of a significant underlying cause. The challenge at the primary healthcare level is differentiating palpitations of a benign aetiology from those related to a significant underlying arrhythmia that requires prompt treatment, investigations and referral.^5,6^

The diagnostic and therapeutic management of this symptom is challenging and can be frustrating for both the patient and the primary healthcare physician, as in many cases a definitive diagnosis of the cause of the palpitations is not reached and no specific therapy is initiated.^6,7^ Many patients will continue to suffer recurrences of their symptoms, which impairs their quality of life both mentally and physically, leading to the risk of adverse clinical events and recurrent visits to the healthcare facilities.

This article reviews the approach of a primary health care physician to patients presenting with palpitations.

## Pathophysiology

The mechanisms responsible for the sensation of palpitations are incompletely understood.^8^ It has been suggested that the neural pathways responsible for the perception of the heartbeat include different structures located at the intracardiac and extracardiac levels.^1^ Palpations usually reflect changes in cardiac rate, rhythm or contractility, and abnormal movement of the heart is felt within the chest.^3^ The patient may be perceiving the augmented post-extrasystoles beat as the ‘skipped’ beat rather than the premature beat itself in case of isolated extrasystoles.^9^

The clinical perception of cardiac phenomena is highly variable. Awareness is heightened in sedentary, anxious or depressed patients and reduced in active, happy patients.^9^ Some patients can be aware of every premature ventricular beat, while others are unaware of even complex atrial or ventricular tachyarrhythmias. In some cases, palpations are perceived in the absence of any abnormal cardiac activity.^9^

## Aetiology

Most palpitations are of cardiac origin, followed by psychiatric and miscellaneous causes such as thyrotoxicosis, caffeine, medication-induced, anaemia, cocaine and amphetamine. In some cases, it is difficult to determine the cause of palpitations.^2,10^

Some patients have heightened awareness of normal cardiac activity, particularly when exercise, febrile illness or anxiety increases the heart rate. However, most cases of palpitations result from arrhythmias. An arrhythmia is defined as any aberrant cardiac rhythm or beat. Arrhythmias range from benign to life-threatening.^9,12^ While arrhythmias often occur spontaneously in patients without serious underlying disorders, others can be caused by a serious cardiac disorder.^9^

The most common (and mostly benign) arrhythmias (Figure 3^11^) include the following^9^:

- Premature atrial contractions (PACs)
- Premature ventricular contractions (PVCs)

Other common arrhythmias include the following^9^:

- Paroxysmal supraventricular tachycardia (PSVT)
- Atrioventricular nodal re-entrant tachycardia
- Atrial fibrillation or flutter
- Ventricular tachycardia
- Bradyarrhythmia’s and heart blocks

Life-threatening causes of palpitations are mostly of cardiac origin and include bradyarrhythmias or tachyarrhythmias. It can be because of atrial causes (e.g. atrial fibrillation or atrial flutter), ventricular causes (e.g. PVCs, ventricular tachycardia and ventricular fibrillation), high output states (e.g. anaemia, pregnancy, sepsis and hyperthyroidism), structural abnormalities (e.g. congenital heart disease, aortic aneurysm, cardiomegaly or acute left ventricular failure) and miscellaneous causes (e.g. postural orthostatic tachycardia syndrome [POTS]).^1,3^ POTS is a cardiovascular autonomic disorder characterised by orthostatic intolerance or inadequate cerebral perfusion on upright posture and is associated with a rapid increase in heart rate.^9^ In patients with orthostatic hypotension, because of an inadequate physiological response to postural changes in blood pressure, the systolic blood pressure decreases by 20 mmHg or the diastolic blood pressure decreases by 10 mmHg within 3 min of standing compared with blood pressure from the sitting or supine position. It commonly leads to palpations on standing.^9^ Ventricular tachycardia and supraventricular tachycardia may present with palpitations, dizziness and/or syncope.^13^

Metabolic conditions that can result in palpitations include hyperthyroidism, hypoglycaemia, hypocalcaemia, hyperkalaemia, hypokalaemia, hypermagnesaemia, hypomagnesaemia and pheochromocytoma.^9^

Anxiety disorders are another common cause of palpitations (Table 1). Information obtained from the patient’s history and family members assists in the diagnosis of anxiety disorder.^11^ Panic disorder is characterised by recurrent unexpected panic attacks. Panic disorder is more common in women of childbearing age.^14^ Environmental stressors may cause persistent palpitations in persons who are highly sensitive to bodily sensations.^15^

**TABLE 1:** Types of palpitations.

Type of palpitations	Subjective description	Heartbeat	Onset and termination	Trigger situations	Potential associated symptoms
Extrasystole	‘Skipping a beat’ ‘Heart sinking’	Irregular, interspersed with periods of a normal heartbeat	Sudden	Rest	-
Tachycardia	‘Beating wings’ in the chest	Regular or irregular, markedly accelerated	Sudden	Physical effort, cooling down	Hemodynamic impairment
Anxiety-related	Anxiety, agitation	Regular, slightly accelerated	Gradual	Stress, Anxiety attacks	Tingling in the hands and face, lump in the throat, atypical chest pain, sighing dyspnoea
Pulsation	Heart pounding	Regular, normal frequency	Gradual	Physical effort	Weakness and lack of strength

*Source*: Adapted from Raviele A, Giada F, Bergfeldt L, et al. Management of patients with palpitations: A position paper from the European Heart Rhythm Association. Eurospace. 2011;13(7):920–934. https://doi.org/10.1093/europace/eur130.^8^

Certain drugs, including digitalis, caffeine, alcohol, nicotine and sympathomimetics (e.g. ibuterol, amphetamines, cocaine, dobutamine, adrenaline, ephedrine, noradrenaline and theophylline), frequently induce palpitations.

Most arrhythmias that cause palpitations have no direct adverse physiologic consequences of their own. However, bradyarrhythmias, tachyarrhythmias and heart blocks can be unpredictable and may adversely affect cardiac output and cause hypotension or death. Ventricular tachycardias, if left untreated, degenerate into ventricular fibrillation.^15^

### Evaluation of a patient with palpitations

A complete history and a physical examination are essential to evaluate a patient with palpitations. Observations by other medical personnel or reliable observers should be sought.^3,9^

## History

A thorough history is important as palpitations are subjective, and the majority of patients will present in sinus rhythm, between episodes of arrhythmia. It is important to clarify the nature of palpitations.^3,9^ Key questions in presenting complaints in history taking are as follows:

- Onset: sudden or gradual
- Duration: momentary or sustained (how long?)
- Frequency
- Triggers
- Associated symptoms:
  ■ Breathlessness
  ■ Chest pain
  ■ Dizziness
  ■ Syncope

## Systems review

Review of systems should cover symptoms of the causative disorder, including heat tolerance, weight loss and tremor (hyperthyroidism); chest pain and dyspnoea on exertion (cardiac ischemia); fatigue, weakness, heavy vaginal bleeding and dark tar-like stools (anaemia); and fatigue, excessive worry, irritability, difficulty in concentrating and sleep disturbances (anxiety disorder)^1,9,16^

## Past medical history

The known potential causes, including documented arrhythmias and heart or thyroid disorders, should be identified. A history of all prescription and over-the-counter medications for example, nasal decongestants, herbal preparations and supplements, such as omega-3 polyunsaturated fatty acids, should be obtained.^4^ Medications used to treat attention-deficit/hyperactivity disorder and reliever inhalers for asthma may cause palpitations.^3,17^ The drug profile should be reviewed for offending prescription drugs (e.g. antiarrhythmics and digitalis).^3^

## Family and social history

Occurrences of syncope or sudden death at an early age should be noted.9 The patient’s social history such as tobacco use, exercise habits, caffeine consumption (including tea and energy drinks), alcohol and illicit drug use should be explored.^4^

The presence of the following red flags should be considered as they increase the possibility of palpitations representing a serious cardiac rhythm disorder^9^:

- Pre-syncope or syncope (particularly if injury occurs from syncope).
- Chest pain or shortness of breath.
- New onset of irregular heart rhythm.
- Heart rate > 120 beats/min or < 45 beats/min while at rest.
- Underlying heart disease.
- Family history of sudden death.

## Examination

### General examination and vitals

The medical practitioner should note if an anxious demeanor or psychomotor agitation is present. Vital signs are reviewed for fever, hypertension or hypotension, tachycardia, bradycardia, tachypnoea or bradypnoea, and low oxygen saturation. Orthostatic changes in BP and heart rate should be measured.^9^

### Peripheral examination

The examination should include inspection of the conjunctivae, palmar creases and buccal mucosa for pallor.^9^ Signs suggestive of hyperthyroidism, such as exophthalmos, thyroid enlargement or tenderness, should be sought.

### Cardiac examination

Cardiac auscultation should note the rate and rhythm as well as any murmurs or extra heart sounds that might indicate underlying valvular or structural heart disease.^9^ However, the examination alone is an unreliable method to determine the arrhythmia causing the palpitations, but certain findings can suggest types of rhythms such as the unique irregular irregularity of some cases of rapid atrial fibrillation, the regular irregularity of coupled atrial or ventricular extrasystoles, the regular tachycardia at 150 beats/min of atrial flutter, which is rare with any other arrhythmia in adults, and the regular bradycardia of < 35 beats/min of complete atrioventricular block.

**FIGURE 1:**
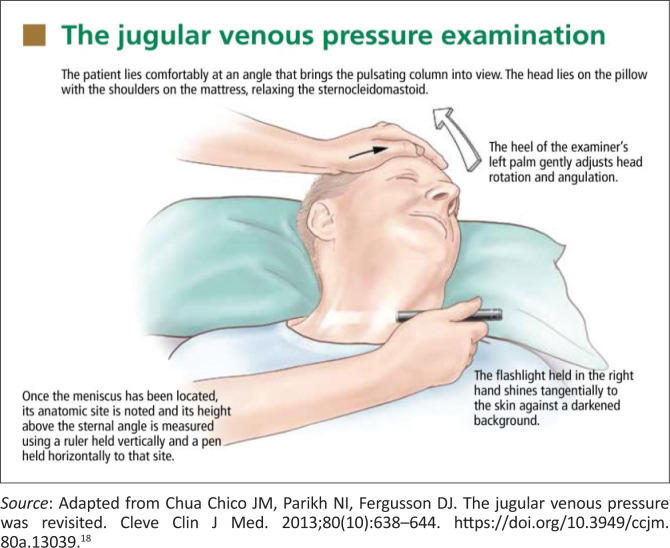
The jugular venous pressure measurement.

**FIGURE 2:**
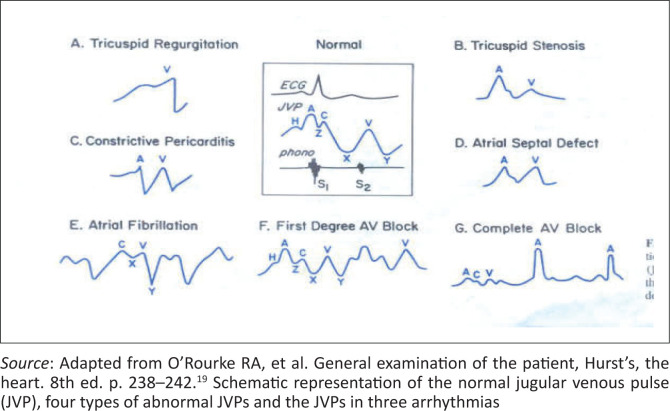
Jugular venous pressure waveform.

Examination of the jugular venous pulse waves is a useful and important element of the physical examination but clinically remains difficult for most practitioners to recognise and interpret (Figures 1 and 2).^17^

**FIGURE 3:**
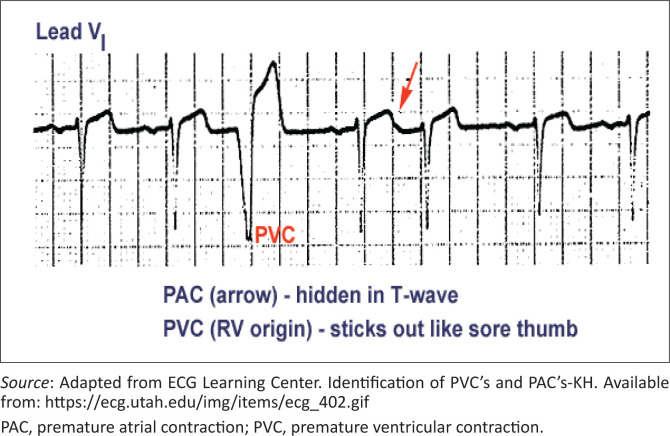
Premature atrial contraction and premature ventricular contraction.

### Neurological examination

Neurological examination should note whether resting tremors or brisk reflexes are present (suggesting excess sympathetic stimulation). An abnormal neurologic finding could suggest that seizures rather than a cardiac disorder may be the cause of syncope and is one of the symptoms.^9^

## Diagnostic evaluation of patients with palpitations

### Electrocardiography evaluation

In all patients who complain of palpitations, a 12-lead electrocardiography (ECG) evaluation is appropriate at the time of symptoms. An ECG is a practical, non-invasive method of recording cardiac rhythm in primary healthcare. Unless the recording is done while symptoms are occurring, it may not provide a diagnosis. Many cardiac arrhythmias are intermittent and show no fixed ECG abnormalities; exceptions include the following:

- Pre-excitation syndromes such as Wolf-Parkinson-White syndrome or Lown-Ganong Levine.
- Long (or short) QT syndromes (LQTS).
- Arrhythmogenic right ventricular dysplasia cardiomyopathy.
- Hypertrophic obstructive cardiomyopathy (HOCM).
- Brugada syndrome and its variants.

Tilt-table testing is a simple, non-invasive diagnostic tool for patients with syncope of unknown origin by inducing syncope.

If no diagnosis is apparent and symptoms are frequent, Holter monitoring, a portable version of an ECG for 24–48 h, is useful for intermittent symptoms, and an event recorder is worn for longer periods.^6^ These tests are used mainly when a sustained arrhythmia is suspected, rather than when symptoms suggest only occasional skipped beats.

Patients with occasional symptoms of palpitations, dizzy spells or syncope where clinicians suspect a serious arrhythmia may have a device implanted beneath the skin of the upper chest (implantable loop recorder [ILR]) by a specialist. The ILR can help diagnose heart rhythm problems that only happen occasionally by continuously recording the rhythm and heart rate and storing them in its memory.

In patients who have palpitations with physical exertion and patients with suspected coronary artery disease or myocardial ischemia, ECG stress testing is appropriate following specialist referral.^3^ Findings from the physical examination or ECG may suggest the need for echocardiography to evaluate structural abnormalities and ventricular function.

### Laboratory testing

Laboratory testing should be limited in primary healthcare to full blood count assessing anaemia and infection, serum urea, creatinine and electrolytes assessing electrolytes and renal function. Thyroid function tests are indicated when atrial fibrillation is newly diagnosed or there are symptoms of hyperthyroidism.^1,9^

Cardiac markers (e.g. troponin and creatinine kinase) should be measured in patients with ongoing arrhythmias, chest discomfort or other symptoms, suggesting recent coronary ischemia, myocarditis or pericarditis.

Patients with findings suggesting cardiac dysfunction, structural heart disease or symptoms on exertion require stress testing, echocardiography and, sometimes, cardiac magnetic resonance imaging (MRI), nuclear scanning or positron emission tomography (PET) and will therefore need to be referred to a tertiary level hospital.^9^

### Management of patients with palpitations

The underlying cause of palpitations determines management.^1^ Precipitating drugs and substances are stopped. If a necessary therapeutic drug causes dangerous or debilitating arrhythmias, a different agent should be tried.^9^ All patients with palpitations associated with red flags might need urgent care by the primary care practitioner and urgent referral to a specialist.

For isolated PACs and PVCs in patients without structural heart disease, simple reassurance and support are appropriate as they are thought to be benign. A medical practitioner should address common risk factors and triggers and promote lifestyle changes to lower stress, stop smoking, and cut back on caffeine and alcohol.^1,9^ For otherwise healthy patients in whom these phenomena are disabling, a β-blocker such as propranolol or metoprolol or calcium channel blocker such as verapamil can be given provided efforts are made to avoid reinforcing the perception of anxious patients who have a serious disorder.^1,9^ Educate select patients with a suspected or documented SVT regarding the use of the Valsalva manoeuvre used to terminate arrhythmias.^3^

Identified rhythm disturbances and underlying disorders are investigated and treated (Table 2).^9^

**TABLE 2:** Some treatments for arrhythmias.

Disorder	Treatment†
**Narrow complex (≤ 120 ms): Tachycardias**	
Multifocal atrial tachycardia	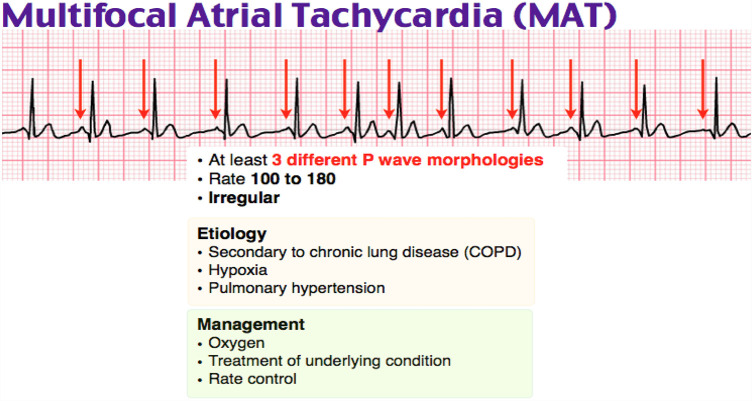 Rate control: β-blockers (verapamil or diltiazem). (Adapted from https://www.google.com/url?sa=i&url=https%3A%2F%2Fwww.grepmed.com%2FimagFmultifocal-tachycardia-ecg-cardiology-diagnosis&psig.)
Atrial flutter	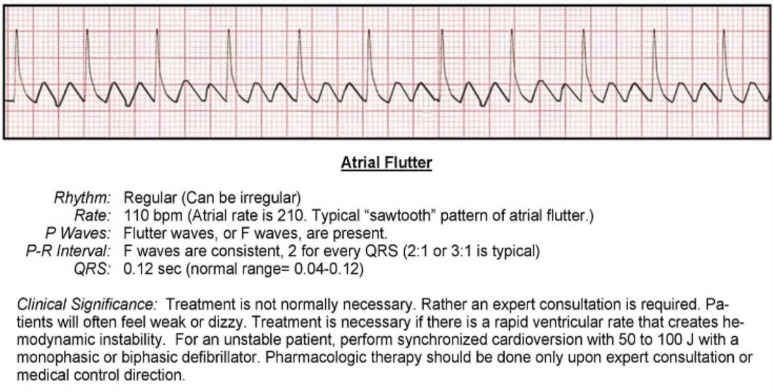
Atrial fibrillation	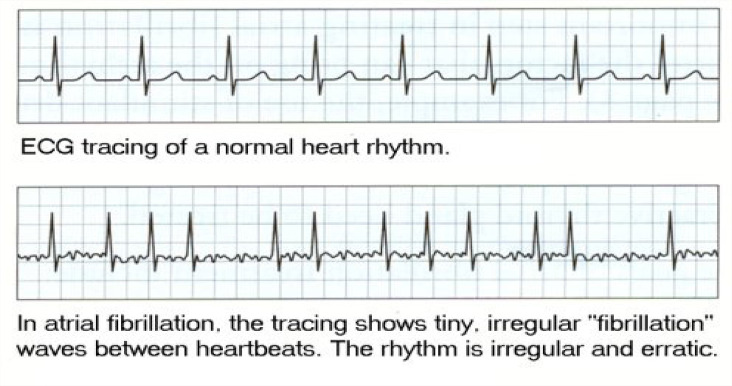 Mx: Stabilise using ABC, direct current (DC) cardioversion for *unstable patients*. Antiarrhythmic drugs may be given before cardioversion with an anticoagulant. For stable patients, rate control with β-blockers or calcium channel blockers and rhythm control with amiodarone can be used plus anticoagulant to prevent thromboembolism. The underlying cause must be identified and corrected.
Supraventricular tachycardia	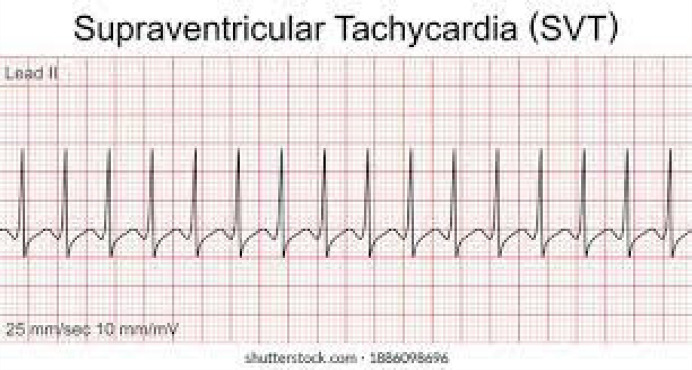 Mx: Vagal stimulation, adenosine or verapamil, or diltiazem may be used. If failed or hemodynamic compromised, synchronised cardioversion is preferred.
Atrioventricular nodal re-entrant tachycardia	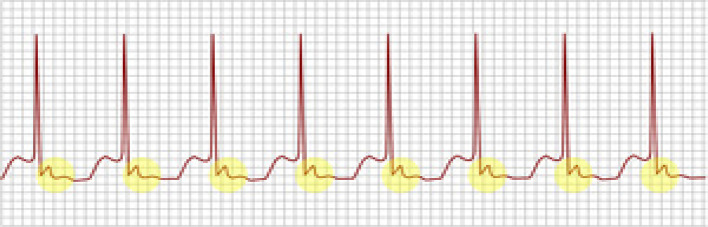 Mx: β-blockers (verapamil or diltiazem), catheter ablation if ineffective.
**Wide QRS complex (≥ 120 ms): Tachycardias**	
Ventricular tachycardia	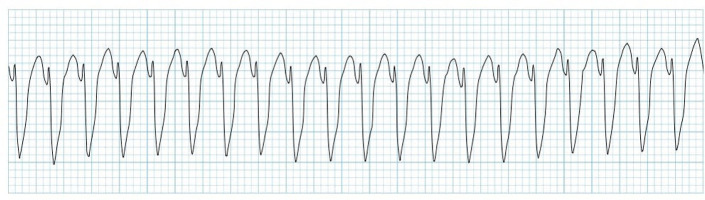 **Stable, monomorphic VT:** Mx: Identify and correct the underlying cause, synchronised cardioversion or anti-arrhythmic, such as amiodarone, sotalol and lidocaine, and once reversion to is sinus obtained, refer to cardiology for consideration for radio-ablation, implanted defibrillator (automated implantable cardiac defibrillator or pacemaker)
Torsade de pointes	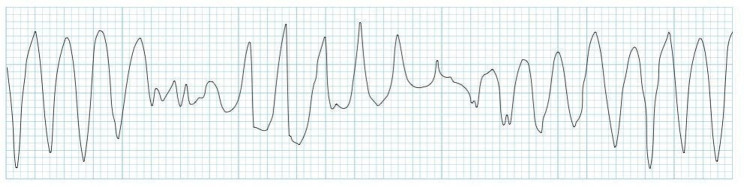 A specific form of polymorphic ventricular tachycardia Mx: Magnesium, potassium, defibrillation, β-blocker, overdrive pacemaker, implanted defibrillator
Ventricular fibrillation, unstable VT	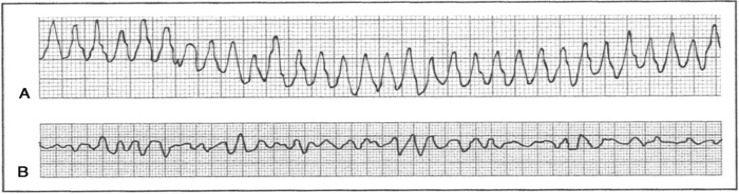 Mx: Defibrillation, β-blocker, amiodarone, lidocaine, implanted defibrillator
Brugada syndrome	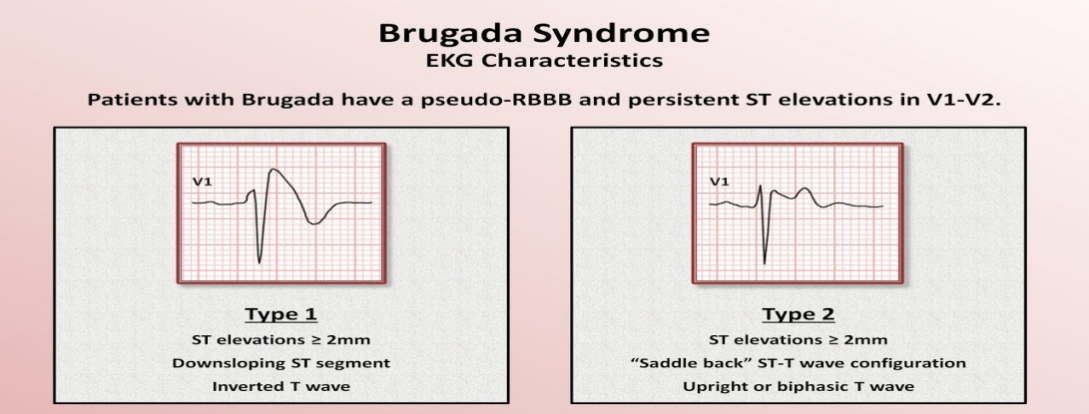 DC, direct-current. DC, direct-current. Mx: Cardioversion, implanted defibrillator, look for underlying trigger (e.g. Infection) that unmasked Brugada

*Source:* Adapted from Thompson A, Shea MJ. Palpitations [homepage on the Internet]. 2020. MSD Manual Profession ed. Available from: www.msdmanuals.com

Note: Vagal stimulation includes Valsalva manoeuvre, carotid massage, ice cold water to the face of a child. Vagal manoeuvres and adenosine injection may help in clinical diagnosis (narrow QRS [Long {or short} QT syndromes {LQTS}] tachycardia), particularly in situations in which the ECG during tachycardia is unclear.

† , Always identity and correct causes and exacerbating factors (e.g. electrolyte abnormalities and hypoxemia, drugs).

## Conclusion

Palpitation is a common symptom occurring in primary care settings. While most are benign, thorough history, examination and awareness of life-threatening conditions can reduce unnecessary referral, and judicious use of limited resources and ECG are essential to exclude serious conditions. Evaluation of a patient presenting with palpitations can be more manageable by understanding the causes and red flags. Standard 12-lead electrocardiography will guide further investigations.

Patients presenting with life-threatening conditions at primary care facilities require immediate intervention and management to stabilise their condition and urgent referral to a higher level of care.

Holter monitor, the portable ECG recording, is a useful tool for diagnosing the causes of occasional palpitations and underlying arrhythmias; it would be beneficial if it were made available in primary healthcare facilities.

Anxiety disorders are a relatively common cause of palpitations in the modern medicine era, and screening of psychiatric aetiology of palpitation should be part of the assessment.
